# D-Limonene Is the Active Olfactory Attractant in Orange Juice for *Bactrocera dorsalis* (Insecta: Diptera: Tephritidae)

**DOI:** 10.3390/life14060713

**Published:** 2024-05-31

**Authors:** Leyuan Liu, Lang Yang, Jinxi Yuan, Jie Zhang, Chenhao Liu, Hongxu Zhou, Wei Liu, Guirong Wang

**Affiliations:** 1 College of Plant Health & Medicine, Qingdao Agricultural University, Qingdao 266071, China; le8825@126.com (L.L.); hxzhou@qau.edu.cn (H.Z.); 2 Guangxi Key Laboratory of Biology for Crop Diseases and Insect Pests/Key Laboratory of Green Prevention and Control on Fruits and Vegetables in South China Ministry of Agriculture and Rural Affairs/Plant Protection Research Institution, Guangxi Academy of Agricultural Sciences, Nanning 530007, China; ylyanglang@gxaas.net; 3 Shenzhen Branch, Guangdong Laboratory of Lingnan Modern Agriculture, Key Laboratory of Synthetic Biology, Ministry of Agriculture and Rural Affairs, Agricultural Genomics Institute at Shenzhen, Chinese Academy of Agricultural Sciences, Shenzhen 518120, China; yuanjinxi1022@163.com; 4 Key Laboratory of Sustainable Management of Forest Ecosystem, Ministry of Education, Northeast Forestry University, Harbin 150040, China; jiezhang666@foxmail.com (J.Z.); liuchenhao98@163.com (C.L.)

**Keywords:** *B. dorsalis*, behavior regulation technology, D-Limonene, odor receptor

## Abstract

The oriental fruit fly, *Bactrocera dorsalis* (Hendel), poses a significant threat to the global fruit industry, causing damage to diverse fruits like citrus, mango, and guava. Chemical pesticides have limited effectiveness, and pesticide residues and pesticide resistance are pressing issues. Therefore, it is essential to develop environmentally friendly pest control methods to address this problem. Behavior-modifying chemicals, including male attractants and intersex protein baits, play a critical role in the control of *B. dorsalis*. The mature host fruit serves as both an oviposition site and food source under natural conditions, making it a potential attraction source for oriental fruit flies. Orange, *Citrus sinensis*, is a main host of *B. dorsalis*, and commercial orange juice is a common attractant for the egg laying of *B. dorsalis*. Although it can both attract and elicit oviposition behaviors in *B. dorsalis* adults, its active components are still unclear. This study utilized analytical chemistry, behavioral tests, and electrophysiology to identify the active components of commercial orange juice that attract *B. dorsalis*, with the aim of providing a reference for the development of behavior-modifying chemical-based techniques to control *B. dorsalis.* Five compounds with a high abundance were identified via a GC-MS, including D-Limonene, butanoic acid ethyl ester, β-myrcene, linalool, and α-terpineol. Behavioral and electrophysiological experiments uncovered that D-Limonene was the active substance that was the main attractant in the mixture of these five substances, evoking a strong electrophysiological response in adult *B. dorsalis*. D-Limonene strongly attracts adult *B. dorsalis* only when they are sexually mature, and the attraction is not rhythmic. Olfaction plays a leading role in the attraction of D-Limonene to adult *B. dorsalis*, and *Orco^−/−^* mediates the perception of D-Limonene by *B. dorsalis*. Overall, D-Limonene is one of the key attractant compounds for *B. dorsalis* in the volatile compounds of commercial orange juice, offering possible support for the development of behavior-modifying chemical-based technology to control *B. dorsalis* in the future.

## 1. Introduction

The fruit fly acts as a significant pest for fruit, causing annual losses exceeding USD 2 billion globally [1]. *Bactrocera dorsalis* is a representative species, having a wide host range and strong adaptability and being difficult to control [2]. It damages over 150 types of fruit, including mangoes, cherries, guavas, loquats, papayas, and citrus fruits, posing a significant threat to agricultural production [3,4]. Since fruit acts as a protective barrier for borer pests, the effectiveness of contact chemical pesticides is limited. The frequent use of chemical pesticides has caused the development of resistance to organophosphorus insecticides, avermectins, and pyrethroids [5,6,7], producing environmental pollution and human health issues.

Semiochemical-based technology is an environmentally friendly pest control method, which employs the relationship between plants and pests mediated by plant volatiles for pest control, leading to the development of various behavior-modifying chemical-based techniques, such as trapping plants and food attractants [8]. Among these techniques, food attractants are biologically based traps that are artificially synthesized and chemically formulated according to the scent of plant stems, leaves, fruits, and other foods. They generally have attractive influences on male and female pests. With the progress in chemical analysis technology and insect olfactory electrophysiological technology, our comprehension of the food odor of pests is constantly deepening. This has caused the development of novel food attractants for pests, including true flies, nocturnal moths, and beetles, through the food volatiles preferred by insects. These attractants have been widely employed globally [1,9].

Behavior-modifying chemicals, including attractants, are important for controlling *B. dorsalis*. Male attractants and intersex protein baits are the most common among these chemicals, producing two control strategies: MAT and BAT [10]. Methyl eugenol is a potent male attractant for *B. dorsalis* that could even eradicate the entire pest population on a small island (e.g., Nauru) [10]. However, its application is limited due to its carcinogenic effect in human cell experiments [11,12] and its inability to control females. Protein baits are less effective than male attractants, but they have also been utilized in the control of fruit flies due to their ability to attract female insects. They can be used in combination with male attractants [10], but they can also attract non-target insects [13]. Therefore, there is an urgent requirement to explore more potential attractant resources to improve the effectiveness of existing attractant compounds.

The host is key as an attractant for fly pests, so it is essential to further explore it as a resource for identifying more active attractant substances. For instance, the host volatiles of *B. cucumis*, *R. pomonella*, *R. mendax*, and *R. cingulata* strongly attract these pests [14,15,16,17], and some of these volatiles have been effective in controlling these pests [18]. *Citrus sinensis* is one of the primary hosts of *B. dorsalis* [19]. Compared to other hosts like guava and papaya, its juice has a strong attraction and oviposition effect on *B. dorsalis* [20]. Additionally, it is often used as a medium for egg collection in the indoor populations of *B. dorsalis* [21]. However, its specific behaviorally active components remain unclear.

In this study, the volatiles of commercial orange juice were isolated using various adsorption methods. These volatiles were then analyzed and identified using gas chromatography–mass spectrometry (GC-MS). Moreover, behavioral tests were performed to identify the active components from the volatile mixtures of the orange. Finally, the candidate receptor of this active substance was characterized using the mutants of olfactory receptor coreceptors. This research offers a scientific foundation for investigating and developing behavior-modifying chemical-based technology to control *B. dorsalis*, aiming to develop a pest control that is environmentally friendly and to reduce the use of chemical pesticides.

## 2. Materials and Methods

### 2.1. Insect Rearing

The experiment utilized four strains of adult *B. dorsalis*, including the wild-type strain, as well as the olfactory co-receptor mutants *Orco^−/−^*, *IR8a^−/−^*, and *IR25a^−/−^* constructed using CRISPR/Cas9 [22]. The insects were maintained in the Shenzhen Institute of Genomics, Chinese Academy of Agricultural Sciences, under conditions of 14 h of light/10 h of darkness, a temperature of 26 ± 1 °C, and a humidity of 60 ± 5%. The larvae were fed an artificial diet composed of bananas, yeast, white sugar, and cellulose paper [21]. Mature larvae were placed in wet sand to pupate and allowed to emerge after pupation. On the third day following the insect emergence, the virgin female and male insects were reared separately in small cages (18 cm × 12.5 cm × 14 cm), with each cage containing 40 insects, alongside water, yeast, and sugar in a 1:1:1 ratio. The insects were starved for 24 h before each behavioral test, during which only water was provided.

### 2.2. Identification of Volatile Compounds by GC-MS

Three methods were employed to obtain the volatile compounds from the orange juice. The first method included dynamic headspace sampling using a simple pull system (HS-P). This system consisted of an air pump, a collection tube (capped with glass wool and filled with Super Q, CNW^®^ technologies, Dusseldorf, Germany), an odor source bottle, a filter tower (containing a molecular sieve and activated charcoal), and a flowmeter. The air stream was controlled at 1.2 L/min, and the collection lasted 24 h. After removing the collection tube, it was rinsed once with 1.5 mL of n-hexane, and the sample was concentrated using a Termovap sample concentrator (Yooning Instrument^®^, Hangzhou, China) and stored at −20 °C. The second method involved static headspace sampling with a solid phase microextraction (HS-SPME) device. For this, 1 mL of orange juice was placed in a 20 mL glass container. SPME Fiber (50/30 µm DVB/CAR/PDMS, Supelco^®^) was employed for the solid-phase microextraction, inserted 4 cm above the orange juice surface and allowed to extract for 20 min. The samples were injected following the extraction. The third method involved the direct injection of headspace (HS-I). In this method, 1 mL of orange juice was directly put into 20 mL headspace vials for direct injection.

The volatile compounds harvested from the orange juice were identified using gas chromatography–mass spectrometry (GC-MS, Thermo, TSQ9000 + trace1310 Agilent, CA, USA) on a DB-5MS column (30 m × 0.25 mm × 0.25 μm film thickness Agilent, CA, USA). The temperature program was as follows: the initial column temperature was 50 °C for 2 min and then heated at 5 °C/min to 290 °C, before being retained for 3 min. The inlet temperature was 250 °C, and the sample size was 1 μL. Helium was employed as the carrier gas at a flow rate of 1.2 mL/min. The mass spectrum analysis conditions were as follows: the ion source was set at EI, and the ion source temperature was set at 300 °C; the electron energy was set at 70 eV, the electron multiplier voltage was set at 350 V, and the quality range was scanned from 50 to 500 *m*/*z*. The chemical compounds in the orange juice were initially identified using computer-standard mass spectrometry (NIST) and manual identification. Subsequently, according to the preliminary identification results, corresponding standards were prepared for re-sampling and verification. The results that were consistent with the standard were considered the final identification results.

### 2.3. Preparation of Commercial Lure and Synthetic Chemicals

Commercial orange juice from Minute Maid ^®^ was employed in the behavioral attractant test. The details of the synthetic chemicals used in this research can be found listed in Table 1. For the GC-MS analysis, each chemical was diluted using n-hexane to a working concentration of 0.01 µg/µL as the analytical standard. For the behavioral tests and electrophysiology, the chemicals were prepared in paraffin oil to produce a stock solution (100 µg/µL), which was then serially diluted to the final working concentrations. The working concentration of the mixtures for the behavior tests and electrophysiology was determined by the ratio of the chromatographic peak areas (the quantitative external standard method) based on the results of the GC-MS. The content of the corresponding chemicals (*M_s_*) in a specific volume of solution was calculated as follows.

*M_s_* = *C_s_* × *V_s_*; *C_s_* = *A_s_* × *C_st_*/*A_st_*

where *C_s_* is the concentration of an individual compound in the sample; *A_s_* represents the peak area of an individual compound in the sample; *C_st_* is the concentration of an individual compound in the standard; *A_st_* denotes the peak area of an individual compound in the standard; and vs. is the total volume of the sample.

### 2.4. Olfactory Trap Assay

Commercial orange juice was employed as an attractant to evaluate whether its volatiles were appealing to adult *B. dorsalis*. According to the response results of the chemical substances in the GC-MS identification, the classes of chemical substances with a high abundance were selected, and the ratio of the compound mixtures (1000 µL) was determined based on the ratio of the chromatographic peak areas (the quantitative external standard method, see Section 2.3). A total of three mixtures was generated, with each mixture removing the compound with the highest abundance to assess the crucial role of this compound in the complex. The active compounds isolated from these experiments were individually tested to characterize their attractiveness on adult *B. dorsalis* across different ages and during varying time periods. Ultra-pure water was employed as the control when assessing the attractiveness of the orange juice. Paraffin oil was used as the control when testing the attractiveness of individual or combined chemicals.

The experiment was performed in insect cages measuring 18 cm × 24 cm × 14 cm. Initially, 30 male insects, aged 12 days, were placed into the cage. The trap consisted of a clear conical flask with a top diameter of 3 cm, a base diameter of 5 cm, and a height of 7.5 cm. An entry point for the adult insects was created using the tip of a 1 mL pipette attached to the flask. Once the adult insects entered the trap, they were unable to escape. Subsequently, two traps were introduced: one containing the attractant (the experimental group) and the other containing paraffin oil (the control group). The two trap bottles were positioned approximately 8–10 cm apart. The cage was positioned under a 50 lux light source for the duration of the experiment. Upon completion, the number of males in each trap was documented. Each experiment was repeated five times following this protocol. The experiment was performed at 26 ± 1 °C from 17:00 to 9:00 the following day. When testing D-Limonene at different ages, the experimental insects were 3 days old, 6 days old, 9 days old, and 12 days old. Adults aged 12 days old were selected to test the attractiveness of D-Limonene at different time periods (9:00–17:00 and 17:00 to 9:00 the following day). The olfactory co-receptor mutants *Orco^−/−^*, *IR8a^−/−^*, and *IR25a^−/−^* were employed to identify the candidate receptors for detecting D-Limonene . Following the experiment, the number of adult flies entering the trap was documented, and the trapping rate was calculated as follows: Attractant rate = (Number of adults in the trap/total number of experimental adults) × 100%.

### 2.5. Electroantennography Recording

Electroantennography (EAG) recordings were performed as described in previous studies [23]. The recordings involved the following steps described below. First, the head of the adult fly was removed, and the end of one of the antennae was excised with fine-tip scissors. Two glass electrodes filled with 0.1 M of KCl were prepared. One electrode was connected to the end of the antenna to record the electrical signals, while the reference electrode was attached to the cut end of the head. The stimulation odor duration was 0.3 s, with an airflow rate of 10 mL/s and an interval of approximately 30 s between the stimuli to ensure the insect antennae activity. A total of 10 adults, all 12 days old, were evaluated. The EAG signals were collected using a preamplifier (Syntech IDAC 4) and further processed using a digital-to-analog converter (IDAC-4 USB System, Syntech, Kirchzarten, Germany). Each odor stimulus was recorded for ten seconds, beginning one second before the stimulus. Offline analysis was conducted using EAG-pro 1.1 software (Syntech, BW, Germany). The final EAG value of the compound was calculated by subtracting the recorded value from the control (which was paraffin oil).

A total of 10 µL of the compound was applied onto a 2.5 cm × 0.9 cm filter paper strip. After drying for 3 min, the liquid was allowed to permeate the filter paper strip without any remaining liquid flowing out. Subsequently, the filter paper strip was placed into the Pasteur tube, sealed, and stored at 4 °C until use. In the EAG experiment involving the primary volatile components in the orange juice, the working concentration of the compound was 10 µg/µL, and the total dose was 100 μg. When comparing the EAG response difference of D-Limonene among the wild type and the olfactory co-receptor mutants *Orco^−/−^*, *IR8a^−/−^*, and *IR25a^−/−^*, the compounds were employed at working concentrations of 0.001–100 µg/µL and doses of 0.01–1000 μg.

### 2.6. Statistical Analyses

All data are presented as mean ± standard error. The original data were initially assessed for normality using the Shapiro–Wilk method. The non-normally distributed data were transformed using log (x + 1). The experimental data were processed by an unpaired *t*-test and a two-way ANOVA. All statistical analyses were conducted using Graph Pad Prism (version 8.0.1).

## 3. Results

### 3.1. Identification of the Main Volatile Components from Orange Juice

**Figure 1:**
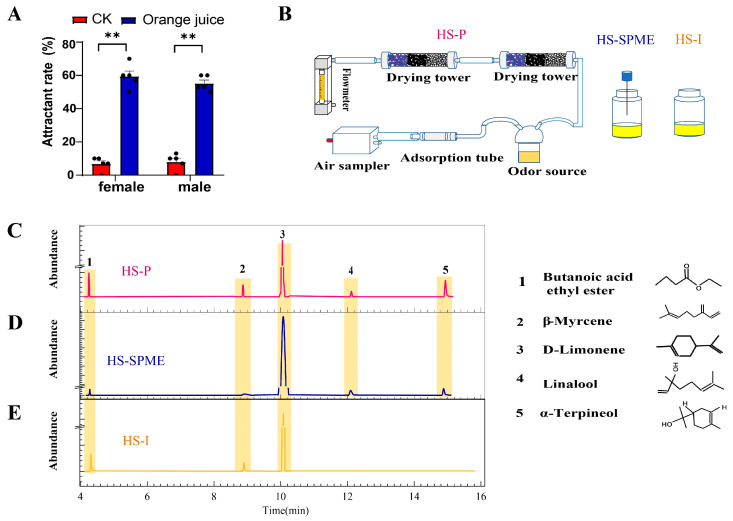
The behavioral response and the identification of volatile compounds from the orange juice. (**A**) The attractiveness of orange juice to both male and female flies. (**B**) schematic drawing of the collection system. (**C**–**E**) The volatiles with high abundance were identified by gas chromatograph-mass spectrometry (GC-MS) utilizing HS-P, HS-SPME, and HS-I sampling methods. (N = 5, ** *p* < 0.01).

The volatile components collected from the orange juice using the methods of HS-P, HS-SPME, and HS-I were identified via a GC-MS. A total of eleven compounds were obtained by the HS-P sampling, and nine were verified by the analytical standard, while seventeen compounds were obtained by the HS-SPME sampling, and eleven were verified by the analytical standard. Additionally, nine compounds were obtained by the HS-I sampling, and eight compounds were verified by analytical standards. The five components with a high abundance (occupying over 1% of the total peak area) were identified as D-Limonene, butanoic acid ethyl ester, β-myrcene, linalool, and α-terpineol (Appendix B, Appendix C, Appendix D). Three mixtures of the compounds mentioned were prepared according to the GC-MS results of the HS-P, HS-SPME, and HS-I. Mixtures 1 and 2 included all five chemicals in different ratios, while Mixture 3 contained only D-Limonene, butanoic acid ethyl ester, and β-myrcene (Figure 1) (Table 2).

### 3.2. Identification of the Behavior-Modifying Components in the Main Volatiles from Orange Juice That Attract B. dorsalis Adults

**Figure 2:**
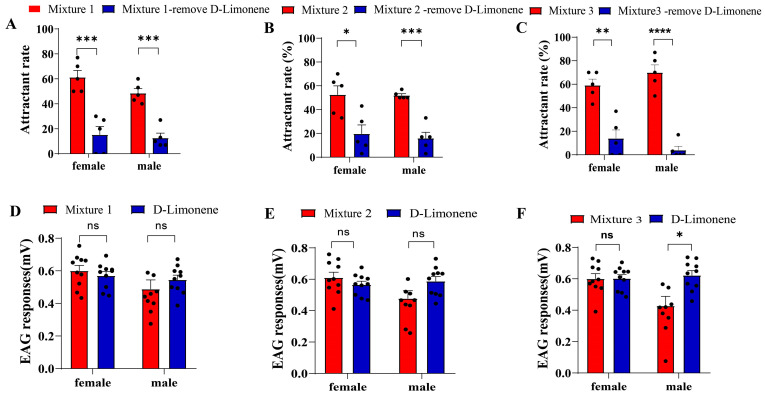
The behavioral and electrophysiological responses produced by the mixtures of chemicals with a high abundance. (**A**–**C**) The attractiveness of combinations with or without D-Limonene. (**D**–**F**) The electrophysiological response of *B. dorsalis* to the mixtures and to D-Limonene by itself (N = 5, ns: not significant, * *p* < 0.05, ** *p* < 0.01, *** *p* < 0.001, and **** *p* < 0.0001).

Using the scent trap experiments, the behavioral activity of three formulations was tested. The findings indicated that both male and female *B. dorsalis* adults could be strongly attracted to the mixture of D-Limonene, butanoic acid ethyl ester, β-myrcene, linalool, and α-terpineol or D-Limonene, butanoic acid ethyl ester, and β-myrcene, with a trapping rate of over 60%. However, following the removal of the component with the highest abundance, namely D-Limonene, the attractiveness of the three mixtures to adult *B. dorsalis* was significantly decreased (Figure 2A, *t*-tests *p* = 0.0012, *p* = 0.0012; Figure 2B, *t*-tests *p* = 0.0135, *p* = 0.0054; and Figure 2C, *t*-tests *p* = 0.0020, *p* < 0.0001). The electrophysiological responses of the identified chemicals to adult *B. dorsalis* were detected via the EAG recording. The results indicated that all mixtures could elicit a strong electrophysiological response from the adult *B. dorsalis*, with the EAG value being over 0.4 mV. When D-Limonene was applied alone, its electrophysiological response achieved the same or a slightly higher intensity than the other components (Figure 2D, *t*-tests *p* = 0.4736, *p* = 0.2943; Figure 2E, *t*-tests *p* = 0.2824, *p* = 0.0695; and Figure 2F, *t*-tests *p* = 0.9720, *p* = 0.0095). In conclusion, D-Limonene plays a key role amongst the orange juice volatiles in attracting *B. dorsalis*.

### 3.3. The Attractiveness of D-Limonene to Adult B. dorsalis of Different Ages and Time Periods and Its Impact on Oviposition Behavior

**Figure 3:**
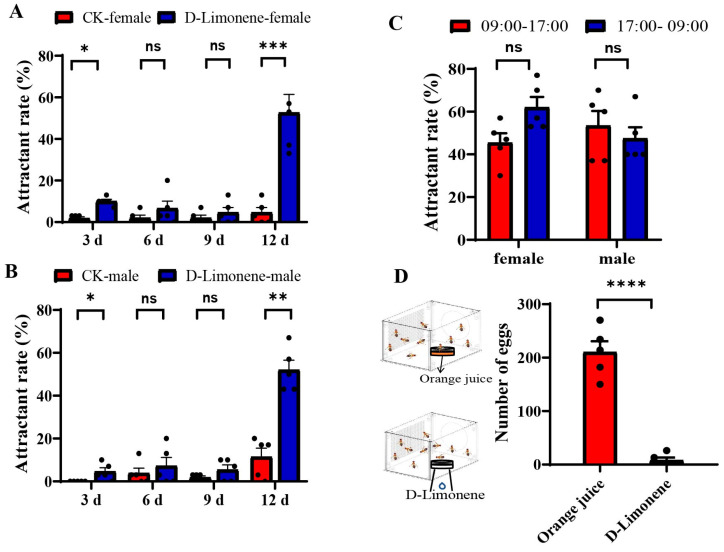
Behavioral responses to D-Limonene. (**A**,**B**) Behavioral responses to D-Limonene by males and females at different ages. (**C**) Behavioral responses of females and males to D-Limonene at different time intervals. (**D**) Comparison of oviposition behavior in females induced by orange juice and D-Limonene. (N = 5, ns: not significant, * *p* < 0.05, ** *p* < 0.01, *** *p* < 0.001, and **** *p* < 0.0001).

Olfactory trap assays were conducted to evaluate the attractiveness of D-Limonene to *B. dorsalis* at different ages during different time periods. The findings revealed that the age of the D-Limonene significantly impacted its attraction to adult flies. At 3 to 9 days of age, D-Limonene exhibited almost no attraction to male and female adult flies, with an attraction rate of approximately 10%. However, at 12 days of age, D-Limonene significantly attracted both male and female flies (Figure 3A,B: a two-way ANOVA where *p* = 0.0002, *p* = 0.0202). Adults at 12 days of age were employed to test the attractiveness of D-Limonene during two different time periods. The finding suggested that the circadian rhythm had no significant effect on the attraction of D-Limonene, as it was capable of attracting adult *B. dorsalis* from 9:00 to 17:00 and from 17:00 to 9:00 the following day (Figure 3C: a two-way ANOVA where *p* = 0.0868, *p* = 0.7066). Given that orange juice is the primary source of egg attraction, the impact of D-Limonene on the egg laying behavior of female flies was evaluated. Compared to the orange juice source, D-Limonene barely induced female flies to lay eggs, with an average number of eggs of 8, compared to 210 with the orange juice source (Figure 3D: a two-way ANOVA where *p* < 0.0001).

### 3.4. The Candidate Olfactory Receptors Mediate the Perception and Behavioral Response of B. dorsalis Adults to D-Limonene

**Figure 4:**
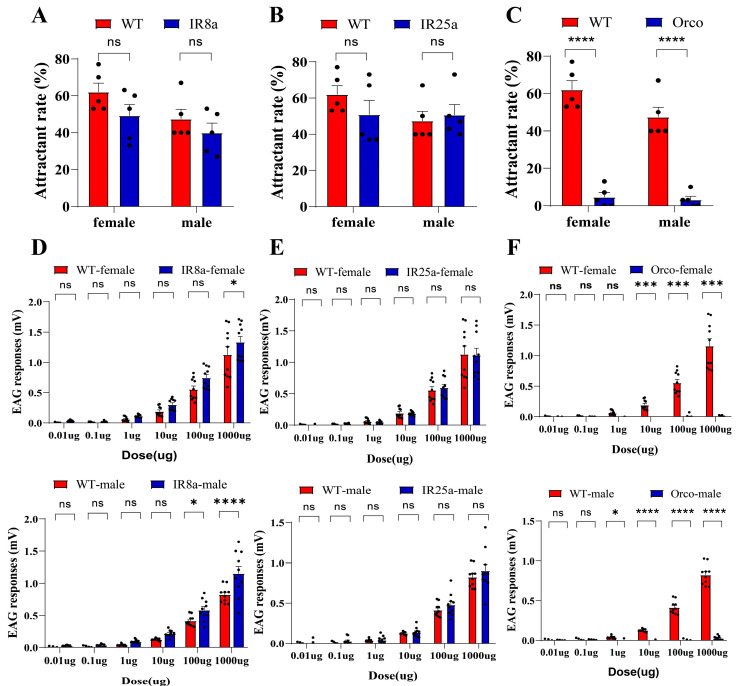
Behavioral and electrophysiological responses of olfactory receptor co-receptor mutants to D-Limonene. (**A**) Behavioral response of *IR8a*^−/−^ to D-Limonene. (**B**) Behavioral response of mutant *IR25a*^−/−^ to D-Limonene. (**C**) Behavioral response of *Orco*^−/−^ to D-Limonene. (**D**) Electrophysiological response of D-Limonene to *IR8a*^−/−^. (**E**) Electrophysiological response of D-Limonene to *IR25a*^−/−^. (**F**) Electrophysiological response of D-Limonene to *Orco*^−/−^ (ns: not significant, N = 5, * *p* < 0.05, *** *p* < 0.001, and **** *p* < 0.0001).

To characterize the olfactory receptor types of *B. dorsalis* responsible for perceiving D-Limonene, we evaluated the behavioral and electrophysiological responses of olfactory co-receptor mutants to D-Limonene. The results indicated that the odorant receptors (ORs) played a significant role in the attraction of D-Limonene to *B. dorsalis*. Compared to the wild-type adults, the *IR8a^−/−^* and *IR25a^−/−^* were strongly attracted by D-Limonene (Figure 4A,B: a two-way ANOVA where *p* = 0.2096, *p* = 0.5695 and *p* = 0.2925, *p* = 0.9051). *Orco^−/−^* adults almost completely lost the behavioral response to D-Limonene (Figure 4C: a two-way ANOVA where *p* < 0.0001, *p* < 0.0001). Using the EAG recordings, the variances in the perception of D-Limonene between the olfactory coreceptor mutants and wild-type adults were contrasted. The results demonstrated that the absence of co-receptor *IR25a^−/−^* and *IR8a^−/−^*, had no significant effect on the electrophysiological response of adults to D-Limonene, and there was no significant difference in the response of the mutants and wild-type adults to this substance over six doses. At high doses, the *IR8a^−/−^* and wild-type adults had different responses to D-Limonene (Figure 4D,E: a two-way ANOVA where *p* = 0.0421, *p* < 0.0001 and *p* > 0.9808, *p* > 0.5077). In the absence of the odor receptor co-receptor *Orco^−/−^*, *B. dorsalis* lost their odor perception of D-Limonene, significantly different from the wild type (Figure 4F: a two-way ANOVA where *p* < 0.001, *p* < 0.0001), indicating that the odor receptor gene exerted a major function in the perception of D-Limonene.

## 4. Discussion

Food attractants founded mainly on host volatiles have become a prominent technique for pest trapping [24]. Food volatiles have behavioral influences on both males and females, with females typically being more attracted. This approach offers unique advantages and significant potential in environmentally friendly pest control technology [25,26]. A single host plant may emit dozens or hundreds of volatiles, but, typically, no more than ten compounds exert a pivotal role. Their composition, proportion, and release level can significantly influence their attractiveness [27,28,29,30]. Therefore, identifying the major components of plant volatiles with behavioral activity is essential for developing food attractants founded on host volatiles. In this study, the volatile components of commercial orange juice were characterized using chemical analysis, and the behavioral activities of the primary responding components were evaluated. Notably, D-Limonene plays a key role in attracting volatile components to both male and female adults. This finding enriched our understanding of the chemical communication between *B. dorsalis* and its host and provided a solid foundation for improving and developing *B. dorsalis* food attractants.

Limonene is an optically active compound composed of two isomers, R. and S. The R-type isomer, D-Limonene, is the primary isomer [31]. Under natural conditions, D-Limonene is widely distributed in the volatile compounds of the fruits and juices of citrus plants, including oranges, lemons, and grapefruits [32,33,34,35]. D-Limonene is a natural product with a limited toxicity to mammals and a high toxicity to beetles and bed bugs. It can be used as a flavoring agent in soaps, perfumes, foods, and some beverages. Additionally, it can be an alternative to synthetic pesticides [36,37,38]. Furthermore, D-Limonene could be a potential behavior-modifying chemical for insects. For instance, it could attract the female potato tuber moth *Phthorimaea operculella* [39]. In this study, D-Limonene was a potent lure for adult *B. dorsalis*, playing a significant role among five orange juice volatiles. Aside from attraction, commercial orange juice can also induce female insects to lay eggs. However, D-Limonene was found to be ineffective in inducing this behavior. Therefore, the active components responsible for oviposition need to be further characterized in the future to develop more effective behavior-modifying chemicals that function on *B. dorsalis*.

Since only chemicals with a high abundance were considered in this study, other chemicals with a high content but a low intensity or low content but strong behavioral responses may have been overlooked. These compounds could be further evaluated using gas chromatography–electroantennographic detection (GC-EAD) to find oviposition attractants or other behavior-modifying compounds. As a polyphagous insect, *B. dorsalis* is attracted to the volatiles of various host species [19,40,41,42,43,44], including linalool and ethyl acetate [45,46]. Therefore, the behavior-modifying compounds from different host sources can be combined with a suitable ratio to enhance the attraction of *B. dorsalis* in the future.

Insects have evolved a highly specialized and sensitive olfactory system capable of detecting environmental changes in order to locate food, mates, and ovipositional sites and to avoid harmful environmental conditions [28,47]. The antennae are the primary chemical organs of insects and are covered with many chemo-sensilla. Olfactory receptors are expressed on the dendrite membrane of the neurons in the olfactory sensilla. These receptors primarily include two types, ORs and IRs, both of which require the participation of a co-receptor to function [48]. In this study, we contrasted the olfactory co-receptor mutant with the wild type of *B. dorsalis* and found that *Orco^−/−^* played a dominant role in both D-Limonene-mediated behavior and adult perception, while *IR8a^−/−^* and *IR25a^−/−^* had no significant effect, indicating that ORs are the main receptors for D-Limonene. Further research could be performed to deorphanize the corresponding ORs by taking advantage of in vitro expression systems, such as *Drosophila* empty neurons [49]. The key OR for D-Limonene could be employed as a molecular target for developing more effective food-based behavioral-modifying chemicals.

The influence of food attractants is closely tied to the physiological condition of insects. All adult flies in this study were deprived of food for 24 h. Therefore, when deploying these chemical compounds, it is essential to thoroughly consider food-related factors, including the pest’s physiological state at the time of application and the presence of competing food sources in the surrounding area. In *Drosophila melanogaster*, hunger can further impact behavior by modulating the perception of food odors in adults. For example, when adults are hungry, reduced insulin levels produce the loss of sNPFR1 inhibition on OR42b neurons in peripheral nerves, amplifying the perception of food odor signals in adults [50]. Consequently, it is crucial to investigate other pivotal genes in the communication mechanism of food source odor perception in *B. dorsalis* in the future, to uncover additional potential strategies for behavioral regulation.

## 5. Conclusions

In this study, we assessed the attractiveness to *B. dorsalis* of the volatiles from orange juice. A GC-MS was employed to analyze the chemical components of these volatiles. Behavioral tests were performed to determine the most crucial behavior-modifying compound among the identified volatile components. We specifically examined the crucial chemical, D-Limonene, to assess its attractiveness to *B. dorsalis* over different ages and time periods, as well as its impact on female oviposition behavior. Olfactory receptor co-receptor mutants were utilized to identify the candidate receptors that respond to D-Limonene. This study deepened our understanding of the role of D-Limonene in locating the host attractant source of *B. dorsalis* and laid the foundation for the future application of D-Limonene in environmentally friendly pest control technologies.

## Figures and Tables

**Table 1:** Compound information required for the experiment.

Compound Name	CAS	Brand Name	State
butanoic acid ethyl ester	105-54-4	Macklin	liquid
D-Limonene	5989-27-5	Macklin	liquid
linalool	78-70-6	Sigma-Aldrich	liquid
α-terpineol	98-55-5	Sigma-AldrichMAC	crystal
β-myrcene	123-35-3	aladdin	liquid
n-hexane	110-54-3	Sigma-Aldrich	liquid
paraffin oil	8012-95-1	Sigma-Aldrich	liquid
α-Pinene	80-56-8	Macklin	liquid
3-Carene	13466-78-9	Macklin	liquid
Terpinen-4-ol	562-74-3	Macklin	liquid
(-)-Carvone	6485-40-1	Macklin	liquid
γ-Terpinene	99-85-4	Macklin	liquid
β-Ocimene	13877-91-3	Macklin	liquid
Cyclohexene, 1-methyl-4-(1-methylethylidene)-	586-62-9	Macklin	liquid

**Table 2:** The ratio and content of the compounds across different mixtures.

Name	D-Limonene	Butanoic Acid Ethyl Ester	α-Terpineol	Linalool	β-Myrcene
Mixture 1	82 (752.3 µg)	12 (110.1 µg)	8 (73.4 µg)	4 (36.7 µg)	3 (27.5 µg)
Mixture 1 without D-Limonene	0	12 (110.1 µg)	8 (73.4 µg)	4 (36.7 µg)	3 (27.5 µg)
Mixture 2	53 (791.0 µg)	6 (89.6 µg)	3 (44.8 µg)	4 (59.7 µg)	1 (14.9 µg)
Mixture 2 without D-Limonene	0	6 (89.6 µg)	3 (44.8 µg)	4 (59.7 µg)	1 (14.9 µg)
Mixture 3	58 (950.8 µg)	2 (32.8 µg)	0	0	1 (16.4 µg)
Mixture 3 without D-Limonene	0	2 (32.8 µg)	0	0	1 (16.4 µg)

**Figure A1:**
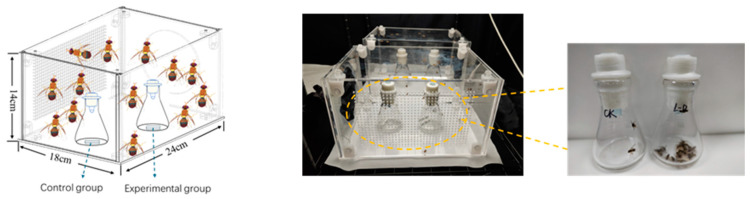
Trap design diagram and actual object diagram.

## Data Availability

The data are included in the main text and Appendix A, Appendix B, Appendix C, Appendix D.

## Appendix B

**Table A1:** The HS-P characterization of the volatile compounds in orange juice.

Component Name	Retention Time	CAS	Peak Area	Area Percentage
D-Limonene	10.092	5989-27-5	385536463	86.6%
butanoic acid ethyl ester	4.565	105-54-4	21978813	4.9%
α-terpineol	14.917	98-55-5	15964525	3.6%
linalool	12.127	78-70-6	6804550	1.5%
β-myrcene	8.935	123-35-3	6236712	1.4%
α-Pinene	7.452	80-56-8	3872509	0.9%
3-Carene	9.478	13466-78-9	1713825	0.4%
Terpinen-4-ol	14.502	562-74-3	1707471	0.4%
Cyclohexene, 1-methyl-4-(1-methylethylidene)-	11.725	586-62-9	1387022	0.3%

## Appendix C

**Table A2:** The HS-SPME characterization of the volatile compounds in orange juice.

Component Name	Retention Time	CAS	Area	Area Percentage
D-Limonene	10.092	5989-27-5	765684109	89.1%
linalool	12.127	78-70-6	16984675	2.0%
α-terpineol	14.917	98-55-5	16539699	1.9%
butanoic acid ethyl ester	4.565	105-54-4	15099217	1.8%
β-myrcene	8.935	123-35-3	11431360	1.3%
(-)-Carvone	16.309	6485-40-1	8960808	1.0%
α-Pinene	7.452	80-56-8	7367255	0.9%
Terpinen-4-ol	14.502	562-74-3	7233630	0.8%
γ-Terpinene	10.913	99-85-4	5046379	0.6%
3-Carene	9.478	99-85-4	4047025	0.5%
Cyclohexene, 1-methyl-4-(1-methylethylidene)-	11.725	586-62-9	861886	0.1%

## Appendix D

**Table A3:** The HS-I characterization of the volatile compounds in orange juice.

Component Name	Retention Time	CAS	Area	Area Percentage
D-Limonene	10.082	5989-27-5	408215296	92.5%
butanoic acid ethyl ester	4.318	105-54-4	15358132	3.5%
β-myrcene	8.902	123-35-3	11328418	2.6%
α-Pinene	7.366	80-56-8	2963893	0.7%
γ-Terpinene	10.917	99-85-4	1780294	0.4%
β-Ocimene	9.462	13877-91-3	949729	0.2%
Cyclohexene, 1-methyl-4-(1-methylethylidene)-	11.716	586-62-9	528556	0.1%
